# When Home Helps or Hurts: A Moderated Mediation Analysis of Work Meaning, Intrinsic Motivation, and Life Satisfaction Across Family Flexibility Profiles

**DOI:** 10.3390/bs15111451

**Published:** 2025-10-24

**Authors:** Tiberiu Dughi, Dana Rad, Alina Roman, Dana Dughi, Camelia Daciana Stoian, Nicolae Radu Stoian, Cristian Măduța, Remus Runcan, Alina Costin, Anca Egerău, Claudiu Coman, Sonia Ignat, Evelina Balaș, Maria Sinaci, Gavril Rad

**Affiliations:** 1 Centre of Research Development and Innovation in Psychology, Faculty of Educational Sciences, Aurel Vlaicu University of Arad, 310032 Arad, Romania; tibi_dughi@yahoo.com (T.D.); dana.dughi@uav.ro (D.D.) remus.runcan@uav.ro (R.R.); alina.costin@uav.ro (A.C.); anca.egerau@uav.ro (A.E.); sonia.ignat@uav.ro (S.I.); evelina.balas@uav.ro (E.B.); gavril.rad@uav.ro (G.R.); 2 Faculty of Humanities and Social Sciences, Aurel Vlaicu University of Arad, 310032 Arad, Romania; camelia.stoian@uav.ro (C.D.S.); cristian.maduta@uav.ro (C.M.); 3 Faculty of Law, Vasile Goldiș Western University of Arad, 310025 Arad, Romania; radustoian73@gmail.com; 4 Department of Social Sciences and Communication, Faculty of Sociology and Communication, Transilvania University of Brasov, 500036 Brasov, Romania; claudiu.coman@unitbv.ro; 5 Centre for Economic Research and Consultancy, Faculty of Economics, Aurel Vlaicu University of Arad, 310130 Arad, Romania; maria.sinaci@uav.ro

**Keywords:** work–life balance, family flexibility, intrinsic motivation, work meaning, life satisfaction, moderated mediation

## Abstract

The present study investigates the twofold effect of home–work spillover on life satisfaction through intrinsic work motivation and meaning derived from work, with family flexibility as a moderator. Based on Self-Determination Theory and the Work–Home Resources model, we test a moderated parallel mediation model whereby both positive and negative spillover from home affect life satisfaction through motivational and meaning pathways, depending on the level of family flexibility. 735 working adults completed validated measures of work-related flow, work meaning, home–work interaction (negative and positive), family flexibility, and life satisfaction. PROCESS macro (Model 59) via 5000 bootstrapped samples indicated that home negatively influencing work was associated with lower life satisfaction, mainly via reduced work meaning, particularly for individuals with low family flexibility. Conversely, positive work–home interaction was associated with higher work meaning and, indirectly, greater life satisfaction, with this effect being stronger when family flexibility was lower. Intrinsic motivation was associated with life satisfaction through mediation only when family flexibility was higher. These results indicate work meaning and family context compensatory and buffering effects on well-being. The research adds to integrative work–life interface models by delineating conditional psychological processes that enable employee flourishing.

## 1. Introduction

In modern companies, the interaction between job and family life has attracted more focus as a major factor influencing employee life satisfaction and well-being. The evolution of flexible and hybrid work models has raised the permeability of the lines separating work from home, therefore enhancing the possible advantages and threats of cross-domain contacts. Home-work interface, as characterized by the degree to which experiences in the home domain affect work results, has been explained in negative and positive terms (ten Brummelhuis & Bakker, 2012; Bakker et al., 2011). Though negative spill-over is capable of disrupting performance, lowering energy levels, and heightening stress, positive spill-over relates to higher levels of motivation, personal enjoyment, and higher levels of subjective well-being (Carlson et al., 2011). To account for how these conflicting pathways influence psychological processes, an approach that integrates motivational and contextual mechanisms is needed.

Despite extensive scholarship on work–life interaction, important questions remain regarding how home influences—both positive and negative—translate into subjective well-being through psychological mechanisms. While existing studies have explored spillover effects broadly, few have examined the dual role of work meaning and intrinsic motivation as parallel mediators, or how these processes are conditioned by family flexibility. This omission leaves a theoretical and empirical gap at the intersection of Self-Determination Theory and the Work–Home Resources model, particularly in hybrid work contexts. Addressing this gap, the present study develops and tests a moderated parallel mediation model to clarify how home dynamics shape life satisfaction. Life satisfaction, a core component of subjective well-being (Diener et al., 1985; Voukelatou et al., 2021), captures individuals’ global cognitive evaluations of their lives. By situating life satisfaction within this broader framework, our study connects work–home dynamics to central debates in the well-being literature.

The Work–Home Resources (W–HR) model provides a comprehensive framework for analyzing the interrelationship between resources and demands in diverse contexts, namely how these factors can either augment or diminish personal resources (ten Brummelhuis & Bakker, 2012; Hill et al., 2001). This strategy holds that home life resources—such as emotional support, shared responsibilities, and good interpersonal relationships—increase an individual’s psychological assets, therefore enhancing their workplace involvement and satisfaction with their job. On the other hand, family demands—such as disagreements, emotional stress, or rigidity—may deplete these resources and lower professional performance or life satisfaction. Emotional states, motivation, and psychological well-being of employees over the long term have been empirically shown to be affected by both types of spillover (Hammer et al., 2005). We use the term work–home spillover to emphasize directional processes (positive and negative influences flowing between home and work), in contrast to the broader concept of work–family balance, which refers to a global appraisal of compatibility across roles. Although some papers use these terms interchangeably, we deliberately distinguish them in order to maintain conceptual precision.

At the same time, Self-Determination Theory (SDT) clarifies that the fulfillment of psychological needs—autonomy, competence, and relatedness—fosters intrinsic motivation and well-being across many spheres (Deci & Ryan, 2000; Gagné & Deci, 2005). People who view their work as self-validating and consistent with their values are more likely to be engaged and to have greater energy and purpose. Workplace environments that enable meeting of these demands are probably going to have positive results including more resilience, dedication, and work performance (Deci et al., 2017). But beyond motivation, the concept of finding meaning in work—conceived as the personal conviction that one’s work is meaningful and purposeful—is becoming a core determinant of life satisfaction (Allan et al., 2016a, 2016b). Engaging in meaningful work contributes to the construction of an integrated life narrative and is related to increased overall well-being, particularly under socioemotional stressors.

We have discovered in research that intrinsic motivation and meaning can both serve as unique yet correlated mediators that link work experiences to well-being outcomes. For instance, Allan et al. (2016a) discovered that employees who reported more meaning at work had less stress and more life satisfaction independent of job demands. Meanwhile, intrinsic motivation has been found to buffer against emotional exhaustion and facilitate adaptive coping (Deci & Ryan, 2000).

While the significance of work and home spill-over has been amply documented, fewer studies have considered the moderating role of family flexibility—namely, the family’s ability to redefine roles, rituals, and expectations to accommodate changing demands (Hill et al., 2001; Capitano et al., 2017). At the W–HR level, flexible family systems serve as facilitating contexts that reinforce the benefit of positive spill-over or inhibit the harm of negative spill-over. For example, a warm and flexible home life can buffer decreased work-related meaning by alleviating strain and maintaining emotional buffers. Conversely, inflexible family dynamics may inhibit one’s ability to recover, further extending the adverse impact of home duties on job performance.

The study conducted by ten Brummelhuis and Bakker (2012) recognizes the impact of contextual factors such as partner support and family flexibility on the within-domain process of resource generation. This corroborates SDT’s postulation that social contexts can fulfill or thwart psychological needs, which in turn affect later motivational processes (Deci & Ryan, 2000; Deci et al., 2017). Specifically, when flexibility within the family is high, the individual may feel more autonomous and competent, and therefore the impact of intrinsic motivation and work meaning on life satisfaction will be stronger.

Although previous studies have tested separate mediation models dealing with motivation or meaning, no empirical research has yet investigated both positive and negative work-home spillover at the same time using parallel mediators, with moderation from contextual variables such as family flexibility. The current study seeks to fill this gap by presenting a moderated parallel mediation model in which Home–Work Interaction, both positive and negative, affects Life Satisfaction through Intrinsic Work Motivation and Meaning Through Work, with Balanced Family Flexibility serving as a moderator in the M to Y paths. We focused moderation on the mediator–outcome pathway because family flexibility is theorized as a contextual amplifier or buffer that shapes whether psychological resources (motivation and meaning) translate into well-being. Alternative structures—such as moderation of the spillover-to-mediator (X→M) path—were considered but not adopted, as these would imply that family flexibility directly alters the formation of motivation and meaning, rather than their effectiveness in predicting satisfaction. This conceptual decision aligns with the Work–Home Resources model, where contextual resources primarily condition the mobilization of personal resources toward outcomes.

It is innovative as well as theoretically based in that it integrates two complementary models—Self-Determination Theory and the Work–Home Resources Model—to test the interplay between motivational and contextual resources in the prediction of well-being.

In light of these considerations, the present study examines how home–work interaction—both positive and negative—shapes employees’ life satisfaction through two central psychological mechanisms: intrinsic motivation and the meaning derived from work. We also explore whether family flexibility conditions these relationships, either by buffering the negative effects of home interference on work, or by amplifying the benefits of positive spillover when contextual resources are limited. Drawing on Conservation of Resources theory (Hobfoll, 2001) and role enrichment theory (Powell & Greenhaus, 2006), we argue that employees may compensate for a lack of family flexibility by relying more strongly on positive spillover to sustain well-being. Building on these theoretical foundations, the following section reviews prior research and positions the current study within this literature.

Building on these theoretical foundations, the following section reviews prior research and positions the current study within this literature.

### Literature Review

The concept of Work–Family Enrichment (WFE) suggests that positive experiences in one domain (e.g., home) can enhance functioning in another (e.g., work) by building personal resources such as energy, resilience, and skills (Roche & Haar, 2020). For instance, intrinsic motivation has been shown to enhance job satisfaction via increased enrichment (Roche & Haar, 2020). Similarly, a meta-analysis revealed a robust, reciprocal relationship between work engagement and WFE, emphasizing the role of motivational processes in boundary crossing (Vadvilavičius & Stelmokienė, 2024).

Positive spill-over from home to work has also been linked to reduced psychological distress, particularly when individuals experience autonomy and effective flexible workplace structures (Yucel & Fan, 2023). Prior research also identified that favorable home-to-work connection is associated with reduced stress and increased job satisfaction, especially among dual-earner couples (Hammer et al., 2005). It is important to distinguish the constructs used in our model from broader concepts such as work–family balance and imbalance. Work–family balance/imbalance refer to the overall appraisal of harmony or conflict between work and family roles as global states (Grzywacz & Carlson, 2007). In contrast, home–work interaction, as measured here, reflects the specific directionality of influence, capturing how home life negatively or positively spills over into work. Thus, our two independent variables (home positively or negatively influencing work) are narrower constructs that capture cross-domain processes rather than global evaluations of balance or imbalance.

Intrinsic motivation, fundamental to Self-Determination Theory (SDT), pertains to participating in activities for their inherent gratification (Deci & Ryan, 2000). It has been shown to reduce work–family conflict and predict greater enrichment (Vo et al., 2022). Recent studies further confirm that intrinsic motivation is negatively related to work–family conflict and positively associated with both WFE and work–family balance (Vo et al., 2022).

Flexible work arrangements (e.g., autonomy over schedule or location) have been consistently associated with better work–family balance and lower distress. However, such flexibility also risks increasing workload if not coupled with clear boundaries (Leslie et al., 2012; Kossek et al., 2012). Moreover, flexible team environments have been found to contribute to improved home–work spill-over and healthier behaviors (Kossek et al., 2012). Family systems literature further argues that family flexibility is a critical contextual moderator: adaptive family routines can buffer negative spill-over and optimize gains from positive spill-over (Allen et al., 2013).

Rarely have studies combined intrinsic motivation, meaning, and family flexibility into a comprehensive mediated model. However, recent contributions have included motivation in models linking positive spill-over to job satisfaction (Roche & Haar, 2020). Based on SDT, supportive contexts are essential for motivational pathways to generate meaningful outcomes (Vo et al., 2022).

Current literature indicates that home–work spill-over affects well-being through motivational and meaningful constructs, moderated by supportive environments. Nevertheless, no empirical study has simultaneously:

- Modeled both negative and positive home–work spill-over;
- Tested intrinsic motivation and work meaning as parallel mediators;
- Examined family flexibility as a contextual moderator.

To fill these gaps, the present research advances both W–HR and SDT frameworks using two moderated parallel mediation models, with balanced family flexibility moderating the mediational pathways for both negative and positive spill-over.

Based on the reviewed literature, we propose the following hypotheses:

**H1.**  *Home negatively influencing work will be associated with lower levels of intrinsic motivation and meaning through work, which in turn will predict lower life satisfaction.*

**H2.**  *Home positively influencing work will be associated with higher levels of intrinsic motivation and meaning through work, which in turn will predict higher life satisfaction. These associations will be stronger for individuals with lower family flexibility, consistent with a compensatory mechanism.*

**H3.**  *Family flexibility will moderate the relationship between the mediators (intrinsic motivation and work meaning) and life satisfaction, such that the indirect effects of home–work interaction on life satisfaction will vary across family flexibility profiles.*

**Figure 1:**
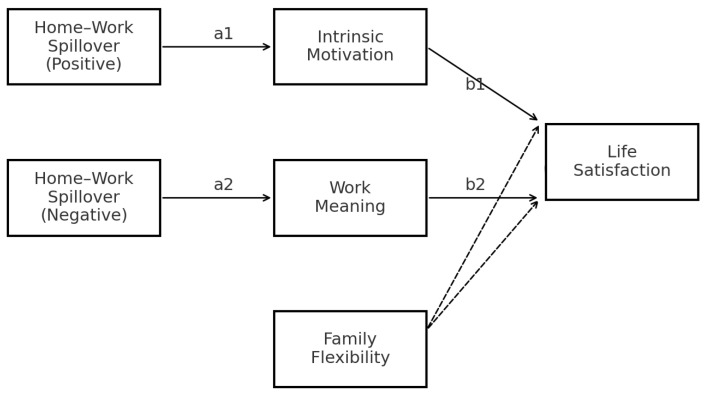
Conceptual model. Family flexibility moderates only the mediator→outcome paths (meaning→life satisfaction; intrinsic motivation→life satisfaction).

Figure 1 presents the hypothesized moderated parallel mediation model. Two dimensions of home–work interaction (positive and negative) are modeled as independent variables. These variables are hypothesized to influence satisfaction with life through two parallel mediators: intrinsic work motivation and meaning through work. Additionally, family flexibility moderates the effects of both mediators on the outcome variable, indicating that the strength of these indirect effects may vary depending on the degree of flexibility in family routines and roles.

Path a1 represents the effect of positive home–work spillover on intrinsic motivation, while path a2 represents the effect of negative home–work spillover on work meaning. Path b1 indicates the effect of intrinsic motivation on life satisfaction, and path b2 indicates the effect of work meaning on life satisfaction. Family flexibility moderates the mediator–outcome paths (b1 and b2), such that the strength of these indirect effects varies across flexibility profiles.

## 2. Materials and Methods

### 2.1. Participants

The study included 735 participants, recruited via convenience sampling from several professional areas in Romania. The predominant demographic of respondents was female, at 90.6%, whilst 9.4% classified as male. This gender distribution is consistent with previous research indicating that women are more represented in professions and academic programs related to education, psychology, and social sciences—fields where emotional labor, caregiving roles, and interpersonal communication are emphasized (Cotton et al., 2013; Jacobs, 1996). Moreover, women are more likely to participate in surveys addressing work–life balance, emotional well-being, and family dynamics, reflecting higher involvement in such domains both professionally and personally (Sullivan & Lewis, 2001).

In terms of educational attainment, 51.8% of participants had completed undergraduate degrees, 41.5% held master’s degrees, and 1.5% had completed doctoral studies. A diminished percentage indicated completion of high school (4.2%) or post-secondary vocational education (1%).

Concerning marital status, 63.4% of participants were married, 18.2% were single, 8.8% were in stable relationships, 8.2% were divorced, and 1.4% were widowed. The majority were engaged in the public sector (86.9%), followed by private sector employees (8.8%), entrepreneurs (2.2%), and freelancers (2.0%).

The majority of respondents indicated over 20 years of work experience (42.9%), followed by those with 11–20 years (23.9%), 1–5 years (14.6%), and 6–10 years (11.6%). A small group (7.1%) had less than one year of experience. A proportion of 12.5% held managerial or leadership positions, while the rest did not report having managerial positions (87.5%) (Table 1).

The participants ranged in age from 18 to 66 years (M = 40.26, SD = 10.84), indicating a broad distribution across early, mid, and late career stages. This variability supports the generalizability of findings across adult working populations.

### 2.2. Instruments

To assess the core constructs in this study, we used validated and culturally adapted instruments, each demonstrating strong internal consistency.

Meaning through work was measured using selected items (2, 7, and 9) from the Work and Meaning Inventory (WAMI) developed by Steger et al. (2012). We opted for this abbreviated version to reduce participant burden, given the extensive survey length, and to capture the most conceptually central items reflecting the purpose and significance of work. To address concerns regarding psychometric integrity, we conducted a confirmatory factor analysis (CFA) which supported the unidimensional structure of the shortened WAMI (χ^2^/df < 3, CFI = 0.95, TLI = 0.94, RMSEA = 0.052). The internal consistency of this shortened scale in our sample was high (Cronbach’s α = 0.88), aligning with the original scale’s reliability estimates (α = 0.84–0.91).

Intrinsic motivation was assessed using the Work-related Flow Inventory (WOLF; Bakker, 2008), specifically the subscale focused on absorption and intrinsic interest in work (Geurts et al., 2005). This subscale reflects the degree to which individuals experience absorption and deep interest in their professional tasks (e.g., “I feel completely absorbed when I am working”). In the current sample, internal consistency was satisfactory (α = 0.85), comparable with previous WOLF applications.

Home–work interaction was evaluated using two subscales from the Survey Work–Home Interaction—Nijmegen (SWING) developed by Geurts et al. (2005): Home→Work Positive and Home→Work Negative spill-over. These subscales measure how the home environment impacts professional life, with sample items such as “A positive atmosphere at home improves my performance at work” (positive spill-over), and “Stress at home affects my concentration at work” (negative spill-over). Both subscales showed excellent reliability (α = 0.92 for positive, α = 0.90 for negative).

Family flexibility was measured using the Balanced Flexibility subscale from the Family Flexibility Evaluation Scale, based on the theoretical model proposed by Hill et al. (2001). This subscale captures how well family roles and routines adapt to changing demands (e.g., “My family adjusts easily when routines or roles change”). Only the balanced flexibility dimension was retained in the analysis. Internal consistency was good (α = 0.83).

Life satisfaction was assessed with the widely used Satisfaction With Life Scale (SWLS) developed by Diener et al. (1985), consisting of five items rated on a 7-point Likert scale (e.g., “In most ways my life is close to ideal”). This scale showed high reliability in our sample (α = 0.87), consistent with its international application.

A CFA supported the measurement structure; detailed fit indices appear in Appendix A Table A1 (χ^2^, df, CFI, TLI, RMSEA, SRMR). Standardized loadings, item-level standard errors, and R^2^, together with construct-level AVE and CR, are presented in Appendix A Table A2. Discriminant validity is reported in Appendix A Table A3 (inter-construct correlations with square-root (AVE) on the diagonal) and Appendix A Table A4 (HTMT with 5000 bootstrap 95% CIs). Harman’s single-factor solution did not account for the majority of variance, reducing concern for common-method bias; results are summarized in Appendix A Table A1.

The CFA demonstrated good fit (χ^2^(344) = 812.4, χ^2^/df = 2.36; CFI = 0.935; TLI = 0.921; RMSEA = 0.052; SRMR = 0.046), supporting the distinctiveness of the constructs. To test for common method variance, we conducted Harman’s single-factor test. Results showed that the first factor accounted for 26.80% of the variance, below the 50% threshold, indicating that common method bias is unlikely to threaten our findings.

All instruments were administered in Romanian. For each subscale, item scores were averaged to compute a mean score. All constructs demonstrated adequate to excellent psychometric properties, supporting their use in subsequent regression and moderated mediation analyses.

### 2.3. Procedure

Data were gathered through an online questionnaire utilizing Google Forms, disseminated by email, academic mailing lists, and social media sites such as Facebook and LinkedIn. A convenience sampling method was utilized, focusing on adult employees from several sectors throughout Romania. The questionnaire comprised demographic inquiries and verified psychometric instruments.

Before commencing the survey, participants received an informed consent statement detailing the study’s goal, the voluntary nature of their participation, and the confidentiality of their responses. Completing the questionnaire was deemed to signify consent.

The research obtained ethical clearance from the Institutional Research Ethics Committee of the associated university. All methods adhered to national data protection rules, including GDPR, and the ethical norms specified in the Declaration of Helsinki (World Medical Association, 2013). Only fully completed surveys (N = 735) were incorporated into the final analysis.

### 2.4. Statistical Analysis

Data analysis was conducted using IBM SPSS Statistics, Version 29.0 (IBM Corp, 2022), in combination with the PROCESS macro, Version 4.2 (Hayes, 2022), to test the hypothesized moderated parallel mediation models. Specifically, we employed Model 59, which allows for the simultaneous estimation of mediation and moderation effects within a single analytical framework. This approach is particularly suitable for examining complex psychological processes in which mediators and moderators interact to influence a key outcome variable. The conceptual model presented in Figure 1 has been revised to ensure consistency with the statistical approach (PROCESS Model 59). Specifically, family flexibility is modeled as moderating the mediator–outcome paths, which is consistent with the analytical testing.

Prior to conditional process analyses, we estimated a confirmatory factor analysis (CFA) of all reflective constructs. Model fit was evaluated with χ^2^/df, CFI, TLI, RMSEA, and SRMR (CFI ≥ 0.95, TLI ≥ 0.95, RMSEA ≤ 0.06, and SRMR ≤ 0.08 or more conservatively ≤ 0.06) (Hu & Bentler, 1999). Convergent validity was assessed via standardized loadings and average variance extracted (AVE ≥ 0.50), and reliability via composite reliability (CR ≥ 0.70). Discriminant validity was examined using the Fornell–Larcker criterion (square-root (AVE) on the diagonal exceeding inter-construct correlations) and the heterotrait–monotrait ratio of correlations (HTMT) with 5000 bootstrap resamples and bias-corrected 95% CIs. We flagged HTMT values ≥ 0.85 (and noted 0.85–0.90 only with theoretical justification). To further probe method bias, we conducted Harman’s single-factor test. Full results are reported in Appendix A Table A1, Table A2, Table A3 and Table A4. CFA and validity statistics (AVE/CR, HTMT with 5000 bootstraps) were obtained in R (lavaan and semTools packages). Conditional process models were estimated in SPSS PROCESS (v4.0).

Two distinct models were tested. In the first model, home negatively influencing work was specified as the independent variable (X), while intrinsic work motivation and meaning through work were treated as parallel mediators (M1 and M2). The moderating variable (W) was family flexibility, operationalized using the balanced flexibility subscale, and the dependent variable (Y) was life satisfaction. In the second model, the structure remained identical, with the exception that the independent variable was home positively influencing work instead of negative.

In both models, the moderation effect was applied to the M→Y paths, enabling the investigation of whether the relationship between each psychological mediator and life satisfaction was conditioned by levels of family flexibility. This allowed us to assess whether the strength of the psychological pathways from work-related resources (intrinsic motivation and perceived meaning) to subjective well-being was amplified or attenuated in the presence of a flexible family environment.

The analyses were performed using 5000 bootstrapped samples to derive bias-corrected 95% confidence intervals for all direct, indirect, and conditional effects. The use of this non-parametric resampling technique provides robust estimates, particularly under conditions of non-normal data distributions, which are common in psychological research. An effect was deemed statistically significant if the corresponding confidence interval did not contain zero.

Further, the models incorporated the estimation of conditional direct effects of the independent variable on life satisfaction at three key levels of the moderator: low (16th percentile), moderate (50th percentile), and high (84th percentile) family flexibility. Additionally, conditional indirect effects (i.e., moderated mediation) were calculated for each pathway involving the mediators, again across varying levels of the moderator. Highest-order unconditional interactions were also examined to determine the presence and strength of moderated mediation effects across the full range of the moderator. Family flexibility was modeled strictly as a moderator of the mediator→outcome paths, namely the links from work meaning and intrinsic motivation to life satisfaction. No moderation was specified or interpreted on the spillover→mediator paths.

To enhance interpretability and reduce multicollinearity, all continuous predictor and moderator variables were mean-centered before the computation of interaction terms.

## 3. Results

### 3.1. Descriptive Statistics and Correlations

Descriptive statistics and bivariate Pearson correlations were computed for all principal study variables. Table 2 displays the means (M), standard deviations (SD), and intercorrelations among the variables: life satisfaction, intrinsic work motivation, meaning derived from work, home-work interactions (both positive and negative), and balanced family flexibility.

Participants indicated elevated life satisfaction (M = 5.76, SD = 0.95), moderate to high intrinsic work motivation (M = 4.94, SD = 1.23), and significant perceived meaning derived from work (M = 4.44, SD = 0.70). Moderate-to-high levels of balanced family flexibility were reported (M = 4.07, SD = 0.61).

Substantial relationships shown in anticipated directions. Life satisfaction exhibited a positive correlation with intrinsic motivation (r = 0.40, *p* < 0.01), meaning derived from work (r = 0.49, *p* < 0.01), the influence of home on work (r = 0.24, *p* < 0.01), and balanced family flexibility (r = 0.47, *p* < 0.01). Conversely, the adverse impact of home on work was inversely correlated with satisfaction (r = −0.35, *p* < 0.01), meaning (r = –0.28, *p* < 0.01), and intrinsic motivation (r = −0.27, *p* < 0.01). All variables were significantly correlated, justifying their inclusion in the proposed mediation and moderation models.

Discriminant validity was supported: in the Fornell–Larcker matrix the square-root (AVE) exceeded all inter-construct correlations (Appendix A Table A3). HTMT values were below 0.85 for all construct pairs; the Work→Home Positive ↔ Home→Work Positive pair—conceptually the closest—remained within acceptable bounds, with the 95% CI from 5000 bootstraps not exceeding 0.865 (Appendix A Table A4).

### 3.2. Model 1: Home Negatively Influencing Work → Mediators → Satisfaction with Life

To investigate whether the effect of home negatively influencing work on life satisfaction is mediated by intrinsic work motivation and meaning through work, and whether these indirect effects are conditional upon levels of balanced family flexibility, a moderated parallel mediation analysis was conducted using PROCESS macro Model 59 (Igartua & Hayes, 2021). The analysis was based on a sample of 735 participants, and all path coefficients were estimated using 5000 bootstrap resamples with bias-corrected 95% confidence intervals.

The regression model predicting intrinsic work motivation revealed a statistically significant overall fit, F(3, 731) = 41.38, *p* < 0.001, with R^2^ = 0.15. However, the predictor home negatively influencing work was not a significant predictor of intrinsic motivation (b = 0.013, *p* = 0.979). The interaction between home negatively influencing work and balanced family flexibility was also non-significant (b = −0.13, *p* = 0.291). Significantly, balanced family flexibility was a substantial positive predictor of intrinsic motivation (b = 0.76, *p* < 0.001). The findings indicate that while negative home–work spillover does not directly diminish intrinsic drive, increased family flexibility independently fosters greater motivation at work.

The regression model forecasting meaning via labor showed more pronounced impacts, achieving statistical significance, F(3, 731) = 62.70, *p* < 0.001, and accounting for 20% of the variance (R^2^ = 0.20). Home’s adverse impact on work strongly predicted diminished levels of perceived meaning (b = −1.25, *p* < 0.001). Importantly, the interaction between home negatively influencing work and balanced family flexibility was also significant (b = 0.24, *p* = 0.001), indicating that the strength of this relationship was moderated by the flexibility present within the family system. Conditional effects analysis indicated that at low levels of balanced family flexibility (16th percentile), home-related interference was significantly correlated with a decrease in meaning (b = −0.42, *p* < 0.001). Nevertheless, when family flexibility was elevated (84th percentile), the adverse effect diminished and ceased to be statistically significant (b = −0.11, *p* = 0.110). The findings indicate that family flexibility may serve as a protective factor, safeguarding individuals’ capacity to derive meaning from their profession despite the presence of home pressures.

The comprehensive outcome model forecasting life satisfaction was significant, F(7, 727) = 66.18, *p* < 0.001, R^2^ = 0.39, signifying that almost 39% of the variance in life satisfaction was elucidated by the predictors and interaction variables. The direct impact of home adversely affecting work on life satisfaction was statistically significant (b = −0.98, *p* = 0.008), indicating that higher home-to-work interference was associated with lower well-being. The significance of meaning derived from work positively and significantly predicted life satisfaction (b = 0.72, *p* = 0.001), but intrinsic work motivation exhibited no significant influence in this model (b = −0.13, *p* = 0.417). The interaction factors between balanced family flexibility and both home negatively impacting work (b = 0.157, *p* = 0.083) and meaning derived from work (b = −0.096, *p* = 0.088) the 95% CI included zero, indicating potential conditionality in the strength of these relationships. Conditional direct effects demonstrated that home negatively influencing work was most strongly associated with lower life satisfaction at low levels of family flexibility (b = −0.44, *p* < 0.001), with a diminished, although still significant effect at elevated levels of flexibility (b = −0.24, *p* = 0.008).

Bootstrap analysis of indirect effects further clarified these relationships. The indirect effect of home negatively influencing work on life satisfaction via intrinsic work motivation was only significant at high levels of balanced family flexibility (84th percentile), with an estimated effect of b = −0.095 and a 95% confidence interval of [−0.167, −0.040]. In contrast, the indirect effect via meaning through work was strongest at low and moderate levels of flexibility. At low balanced flexibility, the indirect effect was b = −0.163, 95% CI [−0.240, −0.092], and at moderate levels, it remained significant (b = −0.080, 95% CI [−0.129, −0.040]). At elevated levels of family flexibility, the indirect effect mediated by work-related meaning was diminished and ceased to be statistically significant (b = −0.030, 95% CI [−0.082, 0.010]).

Table 3 presents the principal pathways from the moderated parallel mediation model analyzed with PROCESS macro Model 59 (Igartua & Hayes, 2021).

The findings indicate a complex pattern: when individuals encounter significant interference from home in their professional lives, their feeling of purpose derived from work is likely to decline—unless they are supported by a very adaptable and flexible family context. Conversely, intrinsic work motivation seems to function as a protective factor solely when familial flexibility is sufficiently elevated to support and enhance motivational processes. Results underline the significance of incorporating contextual modifiers, such as family system adaptation, into models of work–home interaction and psychological well-being.

These results offer insights into how negative home-work interactions diminish life satisfaction, with this effect being partially mediated by a reduction in work-related meaning. Family flexibility serves as a protective buffer, mitigating adverse effects and sustaining meaning and well-being. These findings enhance the Work–Home Resources model and Self-Determination Theory, providing insights for family-supportive workplace policy.

### 3.3. Model 2: Home Positively Influencing Work → Mediators → Satisfaction with Life

To investigate whether the effect of home positively influencing work on satisfaction with life is mediated by intrinsic work motivation and meaning through work, and whether these direct and indirect effects are moderated by balanced family flexibility, we conducted a moderated parallel mediation analysis based on 5000 bootstrapped resamples in a sample of 735 participants.

The model predicting intrinsic work motivation was statistically significant, F(3, 731) = 39.23, *p* < 0.001, R^2^ = 0.14. The direct effect of home positively influencing work on intrinsic work motivation approached statistical significance (b = 0.823, SE = 0.459, *p* = 0.073), suggesting a trend toward a positive relationship. The interaction between home positively influencing work and balanced family flexibility was not significant (b = −0.113, *p* = 0.294), indicating that family flexibility did not moderate this path. However, balanced family flexibility itself was a significant predictor of intrinsic work motivation (b = 0.880, SE = 0.309, *p* = 0.0045), showing that individuals perceiving higher family flexibility also reported stronger intrinsic motivation for work.

The regression model forecasting meaning via work was significant, F(3, 731) = 65.51, *p* < 0.001, R^2^ = 0.21. The positive influence of home on work was a robust and significant predictor of meaning derived from work (b = 0.982, SE = 0.249, *p* < 0.001). The effect was notably attenuated by balanced family flexibility (interaction b = −0.184, SE = 0.062, *p* = 0.003). Conditional effects analyses indicated that at low levels of family flexibility, the impact of home on work meaning was most pronounced (b = 0.38, *p* < 0.001). While the effect remained significant at high levels of family flexibility, it was diminished (b = 0.15, *p* = 0.0015). This indicates a compensatory role of meaning: as family flexibility diminishes, individuals extract greater significance from favorable home-work relationships.

The ultimate model forecasting life satisfaction was significant, F(7, 727) = 58.68, *p* < 0.001, R^2^ = 0.36. The direct impact of home favorably affecting work on life satisfaction was not significant (b = 0.176, SE = 0.330, *p* = 0.595), indicating an indirect-only association. The significance of employment in deriving meaning was a robust predictor of life satisfaction (b = 0.912, SE = 0.223, *p* < 0.001). Balanced family flexibility exerted an independent beneficial influence on satisfaction (b = 0.853, SE = 0.289, *p* = 0.003). Additionally, a notable interaction between job meaning and family flexibility (b = −0.144, SE = 0.061, *p* = 0.017) suggested that the beneficial effect of work meaning on life satisfaction was more pronounced when family flexibility was diminished.

The indirect effects indicate that the pathway from home positively impacting work via intrinsic motivation to life satisfaction was significant at medium and high levels of family flexibility (b = 0.047, 95% CI [0.023, 0.077] and b = 0.047, 95% CI [0.015, 0.089], respectively) and marginal at low levels (b = 0.040, 95% CI [−0.001, 0.092]). The indirect effect through work-related meaning was significant across all levels of family flexibility, exhibiting a diminishing strength: strongest at low levels (b = 0.163, 95% CI [0.093, 0.232]), moderate at medium levels (b = 0.083, 95% CI [0.048, 0.123]), and weakest, yet still significant, at high levels (b = 0.038, 95% CI [0.010, 0.074]).

These findings validate a fully mediated relationship in which favorable home-work interactions improve life satisfaction through the perceived significance of work, especially in less adaptable family settings. Intrinsic motivation also contributes to satisfaction, but its effect emerges primarily when family flexibility is moderate to high.

The present analysis and results (Table 4) demonstrate that home positively influencing work was associated with greater satisfaction with life, primarily through enhancing the perceived meaning of work. The intrinsic motivational pathway is contingent on having a balanced family flexibility profile, while work meaning serves as a consistent and strong mediator. Furthermore, high family flexibility appears to dampen the power of meaning to drive life satisfaction, highlighting a compensatory role of work meaning in less supportive family environments.

These findings advance both the Work–Home Resources (W-HR) model and Self-Determination Theory, emphasizing how positive family dynamics can either support or substitute for psychological drivers of well-being at work.

In conclusion, the findings provide support for our hypothesized model. Specifically, the results confirm the proposed associations between home–work interaction, mediators, and life satisfaction, and they demonstrate the moderating role of family flexibility. Accordingly, the empirical evidence supports all proposed hypotheses.

## 4. Discussion

The current research provides robust evidence that home-to-work spillover, whether negative or positive, significantly shapes employees’ life satisfaction through psychological processes of work meaning and intrinsic motivation, and these effects are contingent upon family flexibility. Specifically, negative home influences were strongly associated with lower perceived meaning through work, especially under conditions of low family flexibility. This finding echoes results from recent studies showing that domestic conflict diminishes employees’ sense of purpose and engagement in work unless buffered by family adaptability (Montgomery et al., 2009; Soomro et al., 2018; Maglalang et al., 2021). Conversely, positive home influences were significantly associated with higher work meaning, and this enhancement persisted across levels of family flexibility—mirroring findings in other contexts where home support enriches employees’ work experience (Richman et al., 2008; Chan et al., 2020; Siu et al., 2010). The effect of home positivity on intrinsic motivation emerged only when family flexibility was moderate to high, consistent with evidence that motivational benefits from home environments depend on supportive home structures (Shah & Asad, 2018; Shin & Kelly, 2013). By contrast, work meaning demonstrated consistent and robust mediation across spillover types. This asymmetry suggests that meaning appears to function as a more stable evaluative lens through which individuals interpret home–work dynamics, while intrinsic motivation seems more sensitive to contextual scaffolding. Meaning may thus reflect deeper existential integration of roles, whereas motivation fluctuates with environmental support—an interpretation consistent with SDT’s distinction between enduring purpose and situational drive.

One possible explanation for this asymmetry is that meaning of work functions as a more stable evaluative lens, grounded in identity and purpose, which transcends short-term contextual variations. In contrast, intrinsic motivation is more context-dependent, fluctuating with family flexibility and daily demands. This suggests that meaning provides a durable psychological resource that anchors well-being, while motivation reflects more immediate evaluations of task enjoyment.

It is important to emphasize that moderation effects emerged exclusively on the mediator→outcome links. Family flexibility shaped how work meaning and intrinsic motivation translated into life satisfaction, but it did not moderate the spillover→mediator associations. The stronger role of work meaning compared to intrinsic motivation may reflect their different psychological functions. Meaning operates as a relatively stable evaluative lens, shaping how individuals interpret and integrate experiences across domains. Intrinsic motivation, by contrast, is more context-dependent, varying with situational cues and resource availability. This distinction helps explain why family flexibility exerted greater influence on the meaning→life satisfaction pathway: flexibility enables individuals to sustain and enact meaning across home and work contexts, whereas motivation remains more contingent. This pattern resonates with our compensatory hypothesis (H2), in which meaning buffered the limitations of motivation under varying family conditions.

Given the cross-sectional nature of our design, these findings should be interpreted as associative relationships rather than causal pathways. Although the moderated mediation model provides a theoretically grounded structure, the temporal ordering of variables cannot be confirmed.

These findings align with Self-Determination Theory (SDT), which asserts that supportive social environments facilitate the fulfillment of autonomy, competence, and relatedness demands, therefore promoting intrinsic motivation (Deci et al., 2017; McConnell & Metz, 2024; Li & Wen, 2019). Furthermore, our findings corroborate the Work–Home Resources (W–HR) model, which posits that events in the home domain can either enhance or diminish psychological resources, hence influencing job performance and overall well-being (Siu et al., 2010; Chen et al., 2009; Sarwar et al., 2021). Notably, this study advances these theories by demonstrating that family flexibility serves as both a buffer and facilitator, moderating the spillover–meaning and spillover–motivation pathways.

From a practical standpoint, the results assert that organizational interventions should aim to enhance not only work design but also employees’ home work–life integration. Employers may provide flexible scheduling, job crafting possibilities, or remote work options to enhance purpose and motivation—strategies that correspond with recent empirical research in remote and hybrid work environments (Bloom et al., 2015; Vyas, 2022; Carr & Jooss, 2023). Concurrently, family-supportive policies such as telework provisions, flexible parental leave, and family counseling services might enhance coping mechanisms and resource availability, hence promoting employee well-being (Birman et al., 2024; Chambel et al., 2023; Feeney & Stritch, 2019). Furthermore, coaching and counseling professionals may assist clients in reframing home experiences as resources for work development, while also cultivating family-level strategies that enhance flexibility and meaning-making.

However, our study has limitations. It relies on cross-sectional, self-report data, which restricts causal inferences and may introduce common method variance (Podsakoff et al., 2003). The Romanian convenience sample restricts the generalizability across many cultures and organizational settings. Future research ought to employ longitudinal designs, potentially integrated within multilevel Structural Equation Modeling (SEM), to assess causality and investigate variability at both individual and family levels. Moreover, experimental and diary investigations could investigate the dynamic daily variations in spillover and meaning processes, hence enhancing theoretical models.

Finally, this study clarifies the complex interaction between home domain experiences and employee well-being by stressing family flexibility as a significant contextual factor. By delineating how positive and negative spillover influence work meaning and motivation differently under varying levels of family adaptability, the findings extend both SDT and the W–HR model. They also offer actionable insights for organizations, families, and practitioners committed to enhancing employee flourishing.

### 4.1. Theoretical Contributions

This study provides robust empirical support for the proposition that work meaning and intrinsic motivation mediate the relationship between home–work spill-over and life satisfaction, with family flexibility acting as a contextual moderator. Specifically, results demonstrated that when the home domain negatively influences work, it significantly diminishes the psychological meaning of work, particularly in low-flexibility family environments. Conversely, positive home-to-work spillover enhances work meaning and, to a lesser extent, intrinsic motivation—an effect more pronounced in families exhibiting higher flexibility. The model confirms theoretical assumptions derived from the Work–Home Resources (W–HR) model and Self-Determination Theory (SDT), which posit that contextual resources and psychological needs interact to predict adaptive outcomes (Deci et al., 2017; Chen et al., 2009).

It is important to note that while PROCESS models suggest directional relations, our cross-sectional design precludes causal inferences. The results should therefore be viewed as supporting theoretical associations consistent with SDT and the W–HR model, rather than establishing definitive causal mechanisms.

Several studies echo these patterns. For example, Montgomery et al. (2009) found that home stressors undermine perceived work purpose, while Richman et al. (2008) reported that family-supportive environments amplify positive work-related affect. Similarly, Mache et al. (referenced through Allen et al., 2013) demonstrated that psychological detachment and meaning-making processes mediate stress-outcome relationships in dual-role adults. Moreover, findings are consistent with research on boundary theory, suggesting that flexible segmentation practices buffer the strain caused by negative spillover (Kossek et al., 2012; Shah & Asad, 2018). The moderating role of family flexibility aligns with the family systems perspective, which conceptualizes flexibility as a core dimension of family functioning that influences emotional regulation and behavioral adaptation (Allen et al., 2013).

This study contributes to the literature by modeling two parallel psychological mediators—intrinsic work motivation and meaning—while also introducing family flexibility as a moderator, an interaction rarely tested empirically. While past studies have emphasized organizational support (Feeney & Stritch, 2019; Chambel et al., 2023) or autonomy (Li & Wen, 2019; McConnell & Metz, 2024), the inclusion of family-based moderators opens new directions in understanding the work–home interface. It extends SDT by highlighting that fulfillment of intrinsic needs is not only dependent on workplace factors, but also on the structural and emotional scaffolding provided by the family. Furthermore, the findings support empirical work indicating that meaning-making is a trans-contextual process, shaped by interpersonal, affective, and ecological factors (Siu et al., 2010; Sarwar et al., 2021).

Our contribution lies in showing that family flexibility functions specifically at the mediator→outcome level, rather than across all pathways, highlighting the conditions under which meaning and motivation shape life satisfaction.

Beyond theoretical contributions, the study also offers practical implications and acknowledges certain limitations, which are elaborated in the following sections.

### 4.2. Practical Contributions

Practical implications of these findings are substantial. Organizations may enhance life satisfaction and reduce burnout not only by redesigning work tasks to promote autonomy and meaning, but also by enabling flexible work arrangements that accommodate diverse family dynamics (Bloom et al., 2015). Family-supportive practices, such as schedule autonomy or parental leave flexibility, can have downstream effects on employees’ psychological engagement and resilience (Vyas, 2022). At the micro-level, career coaching, counseling, and well-being programs should explicitly consider the family ecosystem when addressing motivational deficits or work disengagement (Birman et al., 2024; Carr & Jooss, 2023).

Beyond the workplace, interventions that foster meaning-making across home and work domains can strengthen employees’ ability to transform potential stressors into resources for growth. This includes family counseling services, flexible parental leave structures, and programs that encourage shared responsibility in household management. These measures can buffer the negative effects of spillover and reinforce the positive, ultimately supporting employee flourishing.

Furthermore, the findings suggest that organizations should integrate family-level considerations into broader human resource policies. For instance, hybrid work arrangements and telework provisions can be designed not only for productivity but also to enhance family flexibility. Coaching and professional development programs that explicitly target meaning and motivation as cross-domain resources can ensure that the benefits of positive home-to-work spillover are maximized.

Taken together, these practical contributions highlight the need for organizations to acknowledge and actively manage the interconnectedness of family and work domains. By supporting family flexibility and creating environments where meaning and intrinsic motivation can thrive, organizations can foster sustainable well-being and long-term engagement among employees.

### 4.3. Research Limitations and Future Directions

Despite its contributions, this study has several limitations that should be acknowledged. First, the cross-sectional design precludes causal inference. Although PROCESS macro models with bootstrapping provide robust statistical estimation, longitudinal and experimental designs are required to validate the temporal ordering of relationships. Future studies could also employ multilevel structural equation modeling (SEM) to test these pathways across time and nested work–family systems (Podsakoff et al., 2003).

Second, the exclusive reliance on self-report measures raises potential concerns about common method variance. While additional CMV testing indicated that this was not a major threat, the use of multi-source or objective indicators (e.g., supervisor ratings, peer assessments, or physiological data such as cortisol stress markers) would increase confidence in the findings.

Third, the Romanian convenience sample—largely composed of public sector employees—limits the generalizability of results. Replication in diverse occupational and cultural contexts is necessary to strengthen external validity. Future research should consider dyadic diary designs or experience sampling methods (ESM) to capture daily fluctuations in spillover and meaning processes, while also integrating family-level data to reflect the ecological nature of work–home dynamics.

Moreover, incorporating objective assessments of family routines and behavioral tracking (e.g., time-use diaries) could offer more precise insights into the buffering function of family customs or gender-based differences in boundary management. Under remote and hybrid work conditions, future research should also examine the role of digital boundaries and techno-invasion as emergent factors influencing motivation and well-being.

Finally, the growing body of psychosomatic and gender-oriented research highlights the value of including biopsychosocial markers in occupational mental health studies, particularly among high-risk populations such as individuals with chronic illness (Bondar et al., 2025, 2024). Complementary multidisciplinary research on personality traits (Chiș et al., 2024), emotional regulation in educational contexts (Perasso & Di Giuseppe, 2024), sociomedical interventions (Gavrila-Ardelean & Gavrila-Ardelean, 2017), dyadic validation (Turliuc & Muraru, 2013), employability programs (Gavrilă-Ardelean, 2016), and occupational stress in vulnerable groups (Gavrilă-Ardelean & Moldovan, 2014) further supports the integration of both individual and contextual factors. Advancing in these directions will refine theoretical models such as the W–HR framework and SDT, positioning motivation and meaning as cross-domain, socially embedded resources within modern ecological models of well-being.

## 5. Conclusions

This study offers compelling evidence that the psychological meaning derived from work represents a key mediating pathway linking home experiences to overall life satisfaction. Both negative and positive home–work spillover was associated with employees’ feeling of purpose and intrinsic motivation at work, however these dynamics are greatly influenced by the level of family flexibility. Family flexibility is not merely a peripheral element; it is a crucial contextual feature that may buffer the adverse associations of home stressors or amplify the beneficial associations of positive home support. These findings underscore that associative patterns suggest the importance of a comprehensive ecological approach on employee well-being, encompassing both corporate elements and familial frameworks and practices. The study enhances the understanding of how life pleasure is dynamically formed through the interaction of home, job, and the psychological significance that connects them. The implications reach policy areas, indicating that fostering flexible, supportive family structures is equally essential for worker resilience and engagement as conventional workplace initiatives. While our study provides robust evidence for the associative links among home–work spillover, family flexibility, work meaning, and life satisfaction, the cross-sectional and self-report design prevents causal claims. Future longitudinal and experimental designs are needed to validate the directional assumptions implied by our model. As contemporary work evolves into a hybrid model with indistinct boundaries, these observations provide implementable methods in organizational growth and family policy to improve overall well-being and sustained productivity.

## Figures and Tables

**Table 1:** Demographic characteristics of the sample (N = 735).

Characteristic	Category	*n*	%
Gender	Female	666	90.6
	Male	69	9.4
Education level	High school	31	4.2
	Post-secondary vocational	7	1.0
	Bachelor’s degree	381	51.8
	Master’s degree	305	41.5
	Doctoral degree	11	1.5
Marital status	Married	466	63.4
	In a stable relationship	65	8.8
	Divorced	60	8.2
	Single	134	18.2
	Widowed	10	1.4
Occupational status	Public sector employee	639	86.9
	Private sector employee	65	8.8
	Entrepreneur/business owner	16	2.2
	Freelancer	15	2.0
Work experience	<1 year	52	7.1
	1–5 years	107	14.6
	6–10 years	85	11.6
	11–20 years	176	23.9
	>20 years	315	42.9
Leadership role	Yes	92	12.5
	No	643	87.5

Note. Percentages are based on valid responses (N = 735). Categories are mutually exclusive.

**Table 2:** Means, standard deviations, and intercorrelations for study variables (N = 735).

Variable	M	SD	1	2	3	4	5	6
1. Satisfaction With Life	5.76	0.95	—					
2. Intrinsic Work Motivation	4.94	1.23	0.401 **	—				
3. Home Negatively Influencing Work	1.44	0.49	−0.354 **	−0.266 **	—			
4. Home Positively Influencing Work	2.89	0.67	0.243 **	0.252 **	−0.046	—		
5. Meaning Through Work	4.44	0.70	0.493 **	0.484 **	−0.279 **	0.306 **	—	
6. Balanced Flexibility (Family)	4.07	0.61	0.469 **	0.322 **	−0.219 **	0.229 **	0.390 **	—

Note. M = Mean; SD = Standard Deviation. *p* < 0.01 (2-tailed) for all significant correlations marked with **.

**Table 3:** Summary of direct and indirect effects in the Moderated Parallel Mediation Model (Home Negatively Influencing Work).

Path	b	SE	95% CI	Significance
Home negatively influencing work → Intrinsic Motivation	0.013	0.510	[−0.988, 1.014]	n.s.
Home negatively influencing work → Meaning through Work	−1.25	0.279	[−1.797, −0.699]	***
Intrinsic Motivation → Life Satisfaction	−0.13	0.166	[−0.459, 0.191]	n.s.
Meaning through Work → Life Satisfaction	0.72	0.217	[0.291, 1.141]	***
Home negatively influencing work → Life Satisfaction (Direct Effect)	−0.98	0.367	[−1.698, −0.256]	**

Note. b = unstandardized regression coefficient; SE = standard error; CI = confidence interval; n.s. = not significant; *p* < 0.01 (**), *p* < 0.001 (***).

**Table 4:** Summary of direct and indirect effects in the Moderated Parallel Mediation Model (Home Positively Influencing Work).

Path	b	SE	95% CI	Significance
Home positively influencing work → Intrinsic Motivation	0.823	0.459	[−0.078, 1.723]	† (*p* = 0.073)
Home positively influencing work → Meaning through Work	0.982	0.249	[0.493, 1.472]	***
Intrinsic Motivation → Life Satisfaction	−0.106	0.168	[−0.435, 0.223]	n.s.
Meaning through Work → Life Satisfaction	0.912	0.223	[0.475, 1.349]	***
Home positively influencing work → Life Satisfaction (Direct Effect)	0.176	0.330	[−0.473, 0.824]	n.s.

Note. b = unstandardized regression coefficient; SE = standard error; CI = confidence interval; n.s. = not significant; † = marginal significance (*p* < 0.10); *p* < 0.001 (***).

## Data Availability

Data will be made available on request by the first author and the corresponding authors.

## Appendix A

**Table A1:** CFA overall fit indices and Harman’s single factor test.

Model	χ^2^	df	CFI	TLI	RMSEA	SRMR	Harman’s Single Factor Variance Explained
Measurement CFA	812.4	344	0.935	0.921	0.052	0.046	26.80%

**Table A2:** Standardized loadings, R^2^, AVE and CR.

(a) Item Loadings by Construct			
**Construct**	**Item**	**Std. Loading**	**R^2^**
Intrinsic Motivation	Q9	0.69	0.48
	Q10	0.72	0.52
	Q11	0.71	0.5
	Q12	0.68	0.46
	Q13	0.74	0.55
Work→Home Negative	Q1	0.76	0.58
	Q2	0.79	0.63
	Q3	0.8	0.64
	Q4	0.77	0.59
	Q5	0.74	0.55
	Q6	0.78	0.61
	Q7	0.72	0.52
	Q8	0.7	0.49
	Q9	0.71	0.5
Home→Work Negative	Q10	0.75	0.56
	Q11	0.71	0.5
	Q12	0.74	0.55
	Q13	0.72	0.52
	Q14	0.76	0.58
	Q15	0.73	0.53
Work→Home Positive	Q16	0.7	0.49
	Q17	0.71	0.5
	Q18	0.73	0.53
	Q19	0.74	0.55
	Q20	0.69	0.48
	Q21	0.72	0.52
Home→Work Positive	Q22	0.71	0.5
	Q23	0.72	0.52
	Q24	0.68	0.46
	Q25	0.7	0.49
(b) Construct reliability and validity			
**Construct**	**AVE**	**CR**	
Intrinsic Motivation	0.503	0.832	
Work→Home Negative	0.584	0.926	
Home→Work Negative	0.553	0.879	
Work→Home Positive	0.503	0.857	
Home→Work Positive	0.501	0.776	

**Table A3:** Fornell–Larcker discriminant validity.

Construct	1	2	3	4	5
1. Intrinsic Motivation	0.709				
2. Work→Home Negative	0.315	0.764			
3. Home→Work Negative	0.328	0.537	0.744		
4. Work→Home Positive	0.408	0.298	0.312	0.709	
5. Home→Work Positive	0.377	0.285	0.296	0.803	0.708

**Table A4:** HTMT ratios with bootstrap 95% CIs (5000 resamples).

Construct Pair	HTMT	95% CI Lower	95% CI Upper
Intrinsic Motivation—Work→Home Negative	0.315	0.242	0.391
Intrinsic Motivation—Home→Work Negative	0.328	0.252	0.412
Intrinsic Motivation—Work→Home Positive	0.408	0.313	0.493
Intrinsic Motivation—Home→Work Positive	0.377	0.289	0.465
Work→Home Negative—Home→Work Negative	0.537	0.472	0.599
Work→Home Negative—Work→Home Positive	0.298	0.221	0.374
Work→Home Negative—Home→Work Positive	0.285	0.207	0.359
Home→Work Negative—Work→Home Positive	0.312	0.236	0.389
Home→Work Negative—Home→Work Positive	0.296	0.221	0.37
Work→Home Positive—Home→Work Positive	0.803	0.721	0.865
